# Profound Vagal Tone and Bradycardia Mimicking Asystole: A Resuscitation Case Report

**DOI:** 10.1155/2022/4759950

**Published:** 2022-03-08

**Authors:** J. Mannion, L. Chapman, K. Deasy, N. S. Colwell

**Affiliations:** Cardiology Department, Tipperary University Hospital, Clonmel, Tipperary, Ireland

## Abstract

A 48-year-old man presented with dizziness. When he arrived at the emergency department, he collapsed and became pulseless. Prior to his collapse, he was asymptomatic and now even participated in multiple marathon and ultra-running events per year. However, he previously experienced a vasospastic inferior STEMI eight years prior from cocaine use. As a result, he had an ischaemic cardiomyopathy with LVEF of 45%. He never took any further illicit substances after the STEMI; instead, he changed his lifestyle completely and commenced extreme endurance sports. After one hour of alternations between VF/VT rhythms and asystole, a rhythm check demonstrated a single complex with a corresponding pulse. He had received 12 mg of epinephrine up to that point as per local resuscitation guidelines. Upon diagnosing extreme bradycardia, 2 mg of total atropine administration resulted in ROSC. We theorise that this bradycardia was a result of increased vagal tone as ROSC was quickly achieved following atropine administration.

## 1. Case

### 1.1. Introduction

We present a case of a marathon/ultra-runner with profound vagal tone contributing to bradycardia and mimicking asystole during a resuscitation code. Further, this bradycardia was not responsive to epinephrine and required atropine to obtain return of spontaneous circulation (ROSC).

A 48-year-old man presented to the emergency department (ED) because of an episode of severe dizziness while out jogging with his friend. His symptoms briefly improved en route to the ED, but returned abruptly upon arrival. He experienced syncope and was found to be pulseless. Subsequently, a cardiac arrest call was initiated. His initial rhythm was ventricular tachycardia (VT) which evolved into ventricular fibrillation (VF). Defibrillation was performed, but the patient was “asystolic” and cardiopulmonary resuscitation (CPR) was resumed.

### 1.2. Examination

Examination revealed a fit man in his 40s wearing running clothing which was wet with sweat, but no obvious abnormalities. There was good bilateral air entry with ventilation via bag mask ventilation with head tilt-chin lift, with no evidence of tension pneumothorax.

### 1.3. Past Medical History

His history included an ST elevation myocardial infarction (STEMI) induced by coronary artery spasm secondary to cocaine use at age 33 treated with thrombolysis.

**Figure 1:**
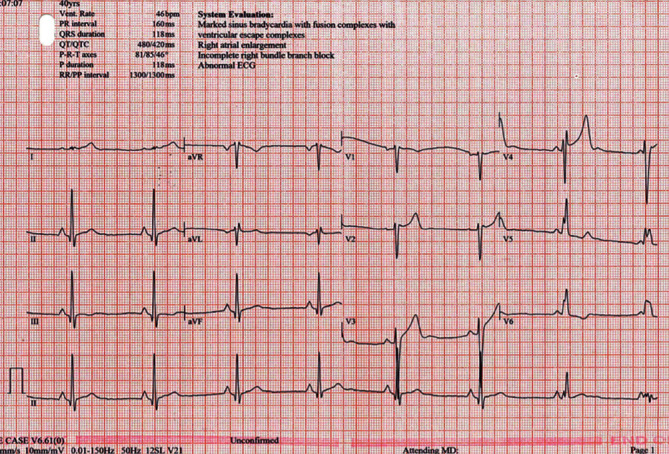
Baseline patient ECG from medical records. Baseline ECG from 2013, with pulse of 46, incomplete RBBB and fusion complexes. We see that our patient had a low resting heart rate in 2013 and has been training for and competing in extreme endurance races such as ultrarunning for the 7 following years.

He had a coronary angiogram 8 years after the initial STEMI with no obstructive coronary disease. His baseline chart ECG was sinus rhythm, rate 46 with an incomplete right bundle branch block (RBBB), with fusion beats (Figure 1).

There was no family history of heart disease. After a normal exercise stress test and cardiology review, he had taken up endurance athletics which became more extreme over time, including marathon and ultrarunning multiple times per year. He quit smoking at the time of his STEMI, having accumulated 18 pack years.

His medications included aspirin 75 mg, ramipril 1.25 mg, and rosuvastatin 20 mg.

Upon discovery of his history 10 minutes into the cardiac arrest, a leading diagnosis of VT/VF secondary to ischaemic scar was made.

### 1.4. Investigations

Initial investigations were conducted during the cardiac arrest. These investigations included rhythm strips, bloods, arterial blood gases, and focused echocardiography.

**Figure 2:**
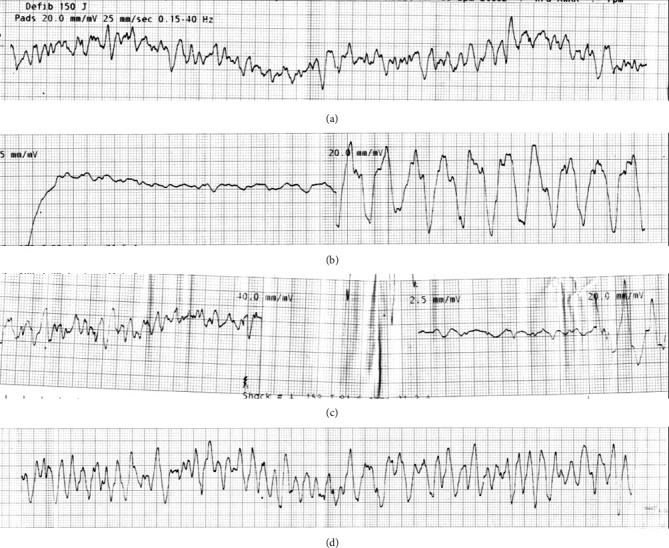
Cardiac arrest rhythm strips. (a) First rhythm strip with ventricular fibrillation identified prior to the first shock. (b) Asystole following defibrillation, this is followed by the arrest team resuming chest compressions. (c) Ventricular fibrillation again during later rhythm check, followed by defibrillation, asystole again, and resumed chest compressions. (d) Further alternation with ventricular fibrillation after prolonged CPR.

*Rhythm strips*: Alternating VF and asystole (rhythm strip A and B, Figure 2)

*Focused arrest echocardiography*: No myocardial hypertrophy, pericardial effusion, tamponade, or right-sided dilation

*Arterial blood gas (20 minutes into CPR)*: pH 7.15 (7.35-7.45), pC02 10.2 kPa (4.6-6.4), p02 11.1 kPa (11.0-14.4), sodium 137 (136-145), potassium 3.0 mmol/L (3.5-5.1), chloride 96 mmol/L (98-107), ionized calcium 1.10 mmol/L (1.15-1.33), glucose 7.2 mmol/L (3.6-5.3), lactate 10.1 mmol/L (0.0–1.3), anion gap 28 mmol/L (10-20), bicarbonate—standard 8.1 mmol/L (21-28), bicarbonate actual 16.1 mmol/L (21-28)

*Full blood count, liver and kidney panel*: WCC 6.5 × 10^9^/L (4-10), neutrophils 2.11 × 10^9^/L (2-7), Hb 13.2 g/dL (13-17), MCV 93.8 fL (83-101), platelets 174 × 10^9^/L (150-400), and CRP < 5; urea, creatinine, electrolytes, coagulation profile, and liver function panel could not be analysed due to haemolysis

*Troponin I*: 0.09 *μ*g/L (<0.04), with repeat at 2 hours 1.28 *μ*g/L

*Urine toxicology*: negative for cocaine, opioids, THC, and amphetamines

### 1.5. Management

Based on his lactic acidosis and possible electrolyte losses, we replaced electrolytes with both 5 g magnesium sulphate and 40 mmol potassium chloride intravenously, in addition to fluid resuscitation during CPR.

**Figure 3:**
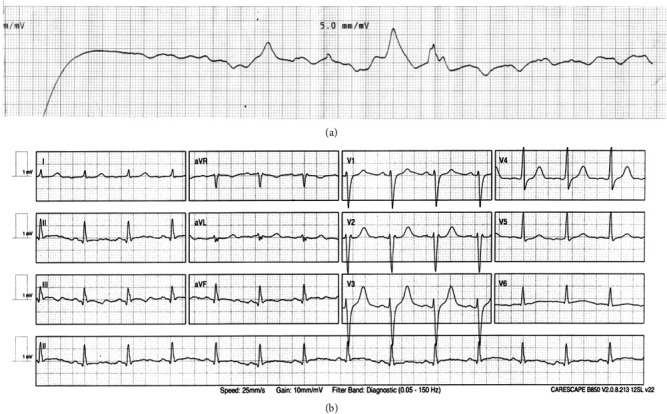
Extended pulse check followed by post-ROSC 12-lead ECG. (a) Rhythm strip with extended observation, demonstrating an intrinsic rhythm, identified complex with corresponding pulse shown with a red arrow. (b) 12-lead ECG of patient with sinus rhythm restored with a rate of 83.

At the one-hour mark, after a further defibrillation and 12^th^ dose of 1 mg epinephrine, a spontaneous complex was sighted during the asystole fortuitously just as CPR was going to resume. A post-shock profound bradycardia secondary to extreme baseline vagal tone and myocardial stunning was suspected and high-dose atropine administered (1 mg followed by another 1 mg when a response was noted). Sinus rhythm with a good rate was restored, and the patient underwent investigations such as chest X-ray and ECG (Figure 3). The patient was transferred to the ICU for post-cardiac arrest care, while on IV antiarrhythmics and ionotropic support to maintain adequate heart rate and mean arterial pressure.

He had a coronary angiogram two days later when extubated and stable, which showed no obstructive disease. He also had an implantable cardioverter defibrillator device implanted for secondary prevention.

### 1.6. Follow-Up

Discharge medications included aspirin 75 mg, bisoprolol 1.25 mg, sacubitril/valsartan 24/26 mg BD, esomeprazole 20 mg, rosuvastatin/ezetimibe 40/10 mg, and paracetamol 1 g QDS/PRN. As an outpatient, he was linked in with our local heart failure service and had a follow-up cardiac MRI which showed an improved LVEF of 38% with no inducible ischaemia on stress perfusion imaging.

## 2. Discussion

Factors such as witnessed collapse, rapid provision of high-quality CPR, initial shockable rhythm, and short duration of resuscitation are good prognosticators for survival. Despite 60 minutes of CPR, our patient had a good outcome. Each case must have the entire clinical picture assessed when deciding on duration of CPR [1].

Post-defibrillation asystole can occur, and in 25% of cases, it can take >120 seconds to return to an organised rhythm [2]. If this was the only factor in our patient's case, we may expect a detectable rhythm during the rhythm check prior to the next shock; however, this was not detected. Animal models of “accentuated antagonism” suggest that chronotropic response to adrenergic stimulation can inversely depend on underlying vagal activity. This may explain our profound response to atropine in the absence of significant epinephrine response [3]. Paradoxical bradycardia in the setting of epinephrine administration is a rare but recognised phenomenon [4].

Evidence suggests that atropine plays no role in an asystole and is managed with epinephrine and good quality CPR. The newest AHA ACLS guidance for symptomatic bradycardia recommends 1 mgas of atropine initial push [5].

## 3. Conclusions

Apparent asystole may not be asystole. Occasionally, profound vagal tone and bradycardia can mimic asystole. This case demonstrates a rare constellation of factors and highlights the phenomenon of profound bradycardia mimicking asystole, as the patient was unresponsive to multiple doses of epinephrine but had rapid clinical improvement following atropine administration.

## Abbreviations

ED: Emergency department
VF: Ventricular fibrillation
VT: Ventricular tachycardia
ROSC: Return of spontaneous circulation
ACLS: Advanced cardiovascular life support
ECG: Electrocardiograph
HFrEF: Heart failure with reduced ejection fraction
HFmrEF: Heart failure with midrange ejection fraction.
